# Validation and Evaluation of a Vendor-Provided Head Motion Correction Algorithm on the uMI Panorama PET/CT System

**DOI:** 10.2967/jnumed.124.267446

**Published:** 2024-08

**Authors:** Fei Kang, Zhaojuan Xie, Wenhui Ma, Zhiyong Quan, Guiyu Li, Kun Guo, Xiang Li, Taoqi Ma, Weidong Yang, Yizhang Zhao, Hongyuan Yi, Yumo Zhao, Yihuan Lu, Jing Wang

**Affiliations:** 1 Department of Nuclear Medicine, Xijing Hospital, Fourth Military Medical University, Xi’an, China; and; 2 United Imaging Healthcare, Shanghai, China

**Keywords:** PET, data-driven, head motion detection, head motion correction, COD, uMI Panorama

## Abstract

Brain PET imaging often faces challenges from head motion (HM), which can introduce artifacts and reduce image resolution, crucial in clinical settings for accurate treatment planning, diagnosis, and monitoring. United Imaging Healthcare has developed NeuroFocus, an HM correction (HMC) algorithm for the uMI Panorama PET/CT system, using a data-driven, statistics-based approach. The HMC algorithm automatically detects HM using a centroid-of-distribution technique, requiring no parameter adjustments. This study aimed to validate NeuroFocus and assess the prevalence of HM in clinical short-duration ^18^F-FDG scans. **Methods:** The study involved 317 patients undergoing brain PET scans, divided into 2 groups: 15 for HMC validation and 302 for evaluation. Validation involved patients undergoing 2 consecutive 3-min single-bed-position brain ^18^F-FDG scans—one with instructions to remain still and another with instructions to move substantially. The evaluation examined 302 clinical single-bed-position brain scans for patients with various neurologic diagnoses. Motion was categorized as small or large on the basis of a 5% SUV change in the frontal lobe after HMC. Percentage differences in SUV_mean_ were reported across 11 brain regions. **Results:** The validation group displayed a large negative difference (−10.1%), with variation of 5.2% between no-HM and HM scans. After HMC, this difference decreased dramatically (−0.8%), with less variation (3.2%), indicating effective HMC application. In the evaluation group, 38 of 302 patients experienced large HM, showing a 10.9% ± 8.9% SUV increase after HMC, whereas most exhibited minimal uptake changes (0.1% ± 1.3%). The HMC algorithm not only enhanced the image resolution and contrast but also aided in disease identification and reduced the need for repeat scans, potentially optimizing clinical workflows. **Conclusion:** The study confirmed the effectiveness of NeuroFocus in managing HM in short clinical ^18^F-FDG studies on the uMI Panorama PET/CT system. It found that approximately 12% of scans required HMC, establishing HMC as a reliable tool for clinical brain ^18^F-FDG studies.

## Visual Abstract

**Figure:**
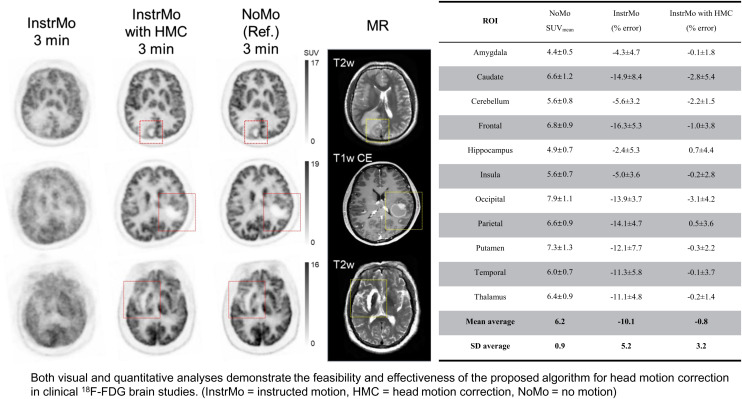

In brain PET imaging, head motion (HM) can lead to errors in uptake estimation and introduce artifacts, compromising diagnostic accuracy. For advanced scanners such as the uMI Panorama PET/CT (United Imaging Healthcare) (1), patient HM significantly hinders achieving the intended spatial resolutions, such as a full width at half maximum of under 3 mm. In clinical settings in which precise quantification is crucial for diagnosis, treatment planning, and response evaluation (2), HM undermines diagnostic confidence. Additionally, HM can cause misalignment between PET and CT images, resulting in attenuation mismatch artifacts and localization issues. In severe cases, substantial HM blurring may necessitate discarding the images and rescanning the patient.

Standard practices to minimize HM during scans include proper patient positioning, clear communication about the procedure, instructions to remain still, sedation, and HM monitoring. However, these measures may not suffice for patients who cannot voluntarily control HM, such as patients with parkinsonian disorders, cognitive impairment, dementia, brain tumors, or neuroinfectious diseases; young pediatric patients; and patients being scanned after trauma or neurosurgery. Therefore, a robust HM correction (HMC) algorithm is highly demanded in clinical practice.

Previous HMC approaches, such as frame-based image registration and hardware-based HM tracking, have their limitations (3–9). Frame-based image registration cannot correct for intraframe HM and attenuation mismatch artifacts. Although hardware-based HM tracking is more accurate and effective (10), its clinical application is hampered by the need to attach a tracking device to the patient, complicating the setup and impacting clinical workflow.

To address these issues, data-driven methods for HMC have emerged as promising alternatives. These techniques, including principal-component analysis (11*,*^12^) and centroid of distribution (COD) (13*,*^14^), estimate rigid HM using PET raw data, offering software-based solutions that integrate seamlessly into routine clinical workflows. A notable advancement is the NeuroFocus algorithm (United Imaging Healthcare), developed for the uMI Panorama PET/CT system, which is equipped with 189-ps time-of-flight resolution and a 35-cm axial field of view. This algorithm, based on a statistics-based method by Revilla et al. (15), detects HM without parameter tuning and differentiates HM-induced COD changes.

This paper presents a 3-fold contribution, first validating the quantitative accuracy of NeuroFocus for the uMI Panorama PET/CT system, then demonstrating the algorithm’s clinical efficacy in diagnosing brain disorders, and finally reporting the frequency and magnitude of HM for the clinical ^18^F-FDG brain studies in this paper. The validation involved a prospective study with 15 volunteers performing instructed HM during PET scans, followed by the application of NeuroFocus on a large clinical cohort of 302 retrospective brain ^18^F-FDG studies. This study is the first to apply an HMC algorithm to a large clinical cohort with short-duration PET acquisitions.

## MATERIALS AND METHODS

### Validation Study Data Acquisition

Fifteen volunteers were enlisted for the prospective validation study, each undergoing a 3-min single-bed-position ^18^F-FDG brain scan (52.2 ± 9.2 min after injection) while instructed to remain still (no HM [NoMo]), followed by another 3-min scan with instructions for substantial translational and rotational HM (instructed HM [InstrMo]). A CT scan for attenuation correction preceded each PET scan. Additionally, T1-weighted, contrast-enhanced T1-weighted, and T2-weighted MR images were acquired for each subject on the same day. The study was approved by the Ethics Committee of the Medical University of Xijing Hospital, Xi’an, China (approval KY-20212145-F-1), which conformed with the revised Declaration of Helsinki (1964). Written informed consent was obtained from all participants.

### Evaluation Study Data Acquisition

The algorithm’s clinical efficacy was evaluated through a retrospective analysis of 302 clinical single-bed-position brain ^18^F-FDG studies, each with a 3.0-min acquisition at 75.0 ± 19.9 min after injection. The studies were categorized as being acquired with no HMC (NMC) or with HMC. Each study included a CT scan for attenuation correction, but no MRI was performed. Detailed patient information is available in Table 1. The institutional review board approved the retrospective study with a waiver of informed consent.

**TABLE 1.** Patient Information for Validation and Evaluation Datasets

Parameter	Validation (with MRI)	Evaluation (without MRI)
Total participants (*n*)	15	302
Mean age ± SD (y)	34.1 ± 13.1	58.2 ± 14.4
Male (*n*)	6	171
Female (*n*)	9	131

### HMC Algorithm

The HMC algorithm consists of 3 steps, that is, HM detection, estimation, and correction. To detect HM, a COD algorithm (14) was used. A COD trace was generated at 1 Hz. By estimating and separating the variation due to count statistics and HM on the COD trace, we could divide the entire study into consecutive HM-free frames (MFFs) separated by the detected HM time points (15). MFFs shorter than 5 s were discarded and excluded from subsequent processing. To estimate and correct the detected HM for MFFs, each MFF was first reconstructed using ordered-subset expectation maximization (OSEM) without attenuation correction. HM estimation was performed by rigidly registering other frames to the reference frame, that is, the first MFF in time. The mutual-information difference was used as the similarity metric. The first MFF was assumed to be aligned with the CT image in space, thus assuming that no HM occurred between the CT and PET acquisitions. The transformation matrix T(*i*) was used to denote the estimate for the *i*th MFF. To generate a matched attenuation map for the *i*th MFF, the CT attenuation map was transformed using the inverse of T(*i*). Subsequently, OSEM (3 iterations × 10 subsets) with attenuation correction was then performed for each MFF using the aligned attenuation map. After reconstruction, the images using OSEM with attenuation correction for all MFFs were transformed back to the reference MFF space using T and were summed to generate the final HMC image. A voxel size of 1.20 × 1.20 × 1.45 mm was used for all reconstructions.

### Evaluation

For the validation dataset, FreeSurfer (16*,*^17^) segmented paired T1-weighted MR images into 109 brain regions of interest (ROIs), which were then resliced to individual PET spaces and were merged into 11 gray matter (GM) regions: amygdala, caudate, cerebellum cortex, frontal lobe, hippocampus, insula, occipital lobe, parietal lobe, putamen, temporal lobe, and thalamus. SUV_mean_ percentage differences between InstrMo and NoMo scans, and between InstrMo with HMC and NoMo, were reported for each GM region.

For the evaluation dataset, brain ROIs were generated via an in-house CT-based segmentation algorithm. After rigid and nonrigid registration with the Montreal Neurological Institute brain MRI template, 116 automated anatomic labeling brain ROIs were warped to individual CT spaces. Cerebellum uptake and SUV_mean_ ratio images were calculated, with a threshold applied to generate a binary GM mask. The cerebellum ROI was used to calculate the cerebellum uptake on the reference frame (first MFF in time) using OSEM with attenuation correction. Additionally, the SUV_mean_ ratio image of the reference frame was calculated using cerebellum uptake as the reference value. A threshold of 1.0 was applied to the SUV_mean_ ratio image to generate a binary GM mask. The intersecting areas between this mask and the 116 ROIs resulted in refined GM ROIs, which were further merged into 11 GM regions according to the automated anatomic labeling definition (18). Supplemental Figure 1 illustrates this ROI generation process (supplemental materials are available at http://jnm.snmjournals.org).

To quantify HM amplitude, the HM distance of each ROI was estimated using image registration. The HM distances of all 116 ROIs for different MFFs were computed first and then averaged per minute. The final HM amplitude for the composite 11 GM ROIs was determined as the average HM distances of the merged sub-ROIs. The maximal HM distance for each case study was also reported.

## RESULTS

### Prospective Validation Study

**FIGURE 1.**
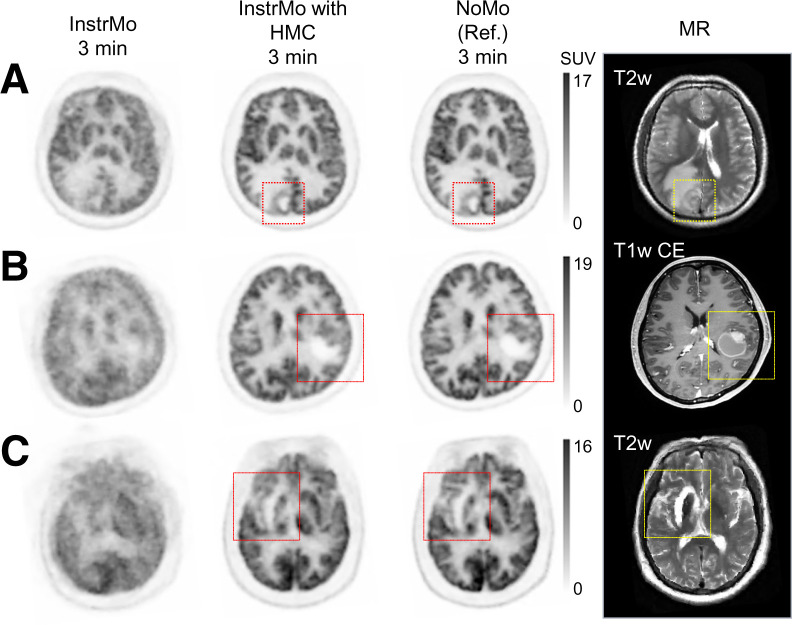
PET and MR images from 3 distinct cases in validation study, comparing InstrMo, HMC, and NoMo. (A) Annular hypermetabolic cerebral syphilitic gumma with surrounding edema in right parietooccipital lobe. (B) Hypermetabolic nodules on PET aligning with nodular wall thickening in cystic-appearing lesion observed on MRI associated with brain metastases from small cell lung cancer. (C) Encephalomalacia and gliosis of right basal ganglia and right temporal lobe and mild *ex vacuo* dilatation of right lateral ventricle in geriatric patient with history of right middle cerebral artery territory infarction. Averaged and maximal HM distance of frontal lobe are 8.5 and 26.3 mm (A), 9.5 and 17.3 mm (B), and 19.0 and 54.2 mm (C), respectively. Injected dose, postinjection time, duration, and body weight are 273.8 MBq, 56.1 min, 3 min, and 69 kg (A); 214.6 MBq, 65.6 min, 3 min, and 56 kg (B); and 270.1 MBq, 67.8 min, 3 min, and 70 kg (C), respectively. T1w CE = T1-weighted contrast-enhanced; T2w = T2-weighted.

In Figure 1, 3 cases from the validation study are shown. Overall, InstrMo images displayed substantial HM-introduced image blur. However, after HMC, these images exhibited marked improvements in both contrast and resolution, closely resembling the NoMo studies. Additionally, the accompanying MR images provided a clear visualization of anatomic structures corresponding to the NoMo studies. Detailed clinical diagnoses for these 3 cases are provided in the caption of Figure 1.

**FIGURE 2.**
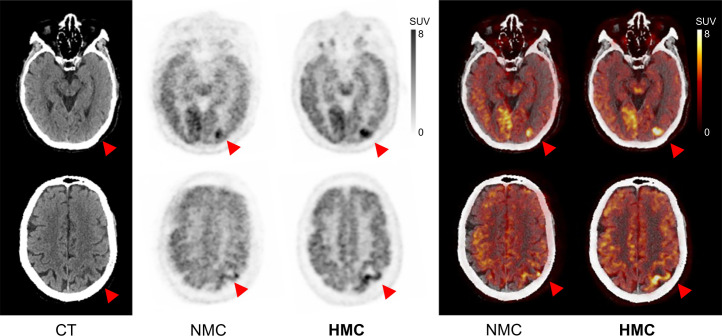
CT, PET, and PET/CT with HM before and after HMC in case of angioimmunoblastic T-cell lymphoma with suspected cerebral infiltration due to acute onset of neurologic symptoms. HM blur and misregistration were corrected after HMC. Areas of annular hypermetabolism with central hypometabolism in left parietal lobe (arrowheads) were revealed after HMC. Injected dose was 251.6 MBq, postinjection time was 90 min, frame duration was 3 min at single bed position, and body weight was 65 kg. Averaged and maximal HM distance of frontal lobe was 10.9 and 18.8 mm, respectively.

Figure 2 presents a patient from the evaluation study diagnosed with angioimmunoblastic T-cell lymphoma. This figure includes 2 axial slices each from the CT, NMC, and HMC images. Notably, the HMC images revealed areas of annular hypermetabolism with central hypometabolism in the left parietal lobe. These areas were largely obscured and indistinct in the NMC images because of the blurring effects of HM.

**FIGURE 3.**
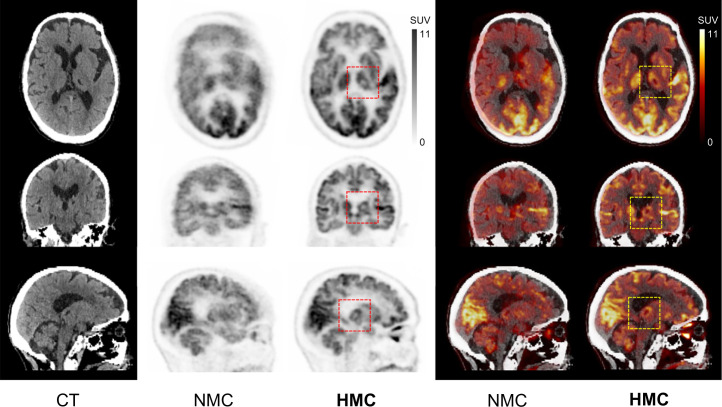
CT, PET, and PET/CT with HM before and after HMC in case of focal hypometabolism observed in left thalamic and basal ganglia region after HMC. Injected dose was 229.4 MBq, postinjection time was 69 min, frame duration was 3 min, and body weight was 48 kg. Averaged and maximal HM distance of frontal lobe was 13.7 and 25.4 mm, respectively.

In Figure 3, a case is shown in which focal hypometabolism in the left thalamic and basal ganglia regions was evident in the HMC image. Conversely, in the NMC image, extensive HM obscured these regions of hypometabolic activity, rendering them invisible.

Numeric analysis of the 15 validation studies is provided in Table 2, showcasing the SUV_mean_ percentage error results for each ROI. The InstrMo scans generally yielded substantial negative differences (−10%), with large variation across different brain regions when compared with the NoMo scans. For instance, the frontal region exhibited a larger discrepancy (−16%) than did the amygdala. The results after HMC showed much smaller differences (∼−1%), with a notably reduced variation (3%), indicating effective compensation for HM in all validation studies.

**TABLE 2.** Percentage Error in SUV_mean_ in Validation Study as Compared with NoMo Study

	Percentage error		
ROI	NoMo SUV_mean_	InstrMo	InstrMo with HMC
Amygdala	4.4 ± 0.5	−4.3 ± 4.7	−0.1 ± 1.8
Caudate	6.6 ± 1.2	−14.9 ± 8.4	−2.8 ± 5.4
Cerebellum	5.6 ± 0.8	−5.6 ± 3.2	−2.2 ± 1.5
Frontal lobe	6.8 ± 0.9	−16.3 ± 5.3	−1.0 ± 3.8
Hippocampus	4.9 ± 0.7	−2.4 ± 5.3	0.7 ± 4.4
Insula	5.6 ± 0.7	−5.0 ± 3.6	−0.2 ± 2.8
Occipital lobe	7.9 ± 1.1	−13.9 ± 3.7	−3.1 ± 4.2
Parietal lobe	6.6 ± 0.9	−14.1 ± 4.7	0.5 ± 3.6
Putamen	7.3 ± 1.3	−12.1 ± 7.7	−0.3 ± 2.2
Temporal lobe	6.0 ± 0.7	−11.3 ± 5.8	−0.1 ± 3.7
Thalamus	6.4 ± 0.9	−11.1 ± 4.8	−0.2 ± 1.4
Mean average	6.2	−10.1	−0.8
SD average	0.9	5.2	3.2

### Retrospective Evaluation Study

**FIGURE 4.**
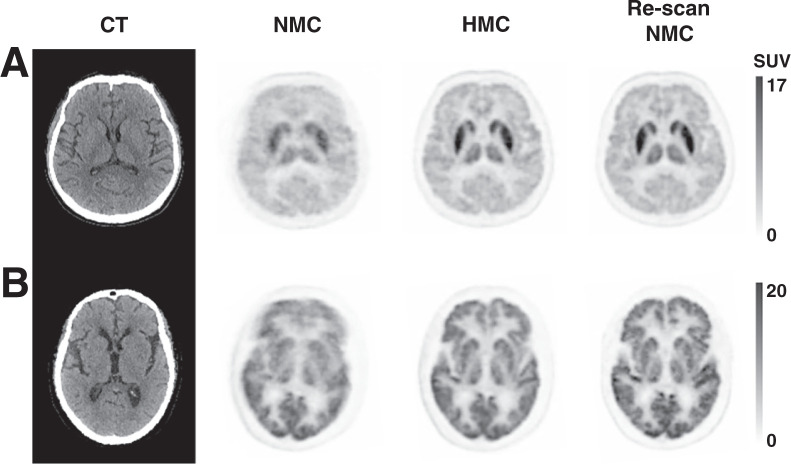
Comparison between PET with NMC, PET with HMC, and PET rescan with minimal HM. (A) Bilateral hypermetabolism in thalami and striatum in patient with suspected nervous system lymphoma. Injected dose was 266.4 MBq, postinjection time was 71 min, frame duration was 3 min, and body weight was 65 kg. (B) Hypometabolic foci in left thalamus indicating lacunar infarcts. Both HMC images are comparable to rescan images. Injected dose was 366.3 MBq, postinjection time was 66 min, frame duration was 3 min, and body weight was 80 kg. Averaged and maximal HM distance of frontal lobe was 9.6 and 47.2 mm (A) and 10.0 and 16.0 mm (B), respectively.

Figure 4 illustrates 2 clinical evaluation studies involving patients with suspected nervous system lymphoma and thalamic lacunar infarction. During the initial PET scans, large HM was detected, prompting the technician to recall the patients for rescanning. Both patients remained still during these subsequent scans. Remarkably, the HMC applied to the initial scans produced images comparable to the rescans, effectively demonstrating the clinical utility of HMC in reducing the need for additional scans due to HM.

**FIGURE 5.**
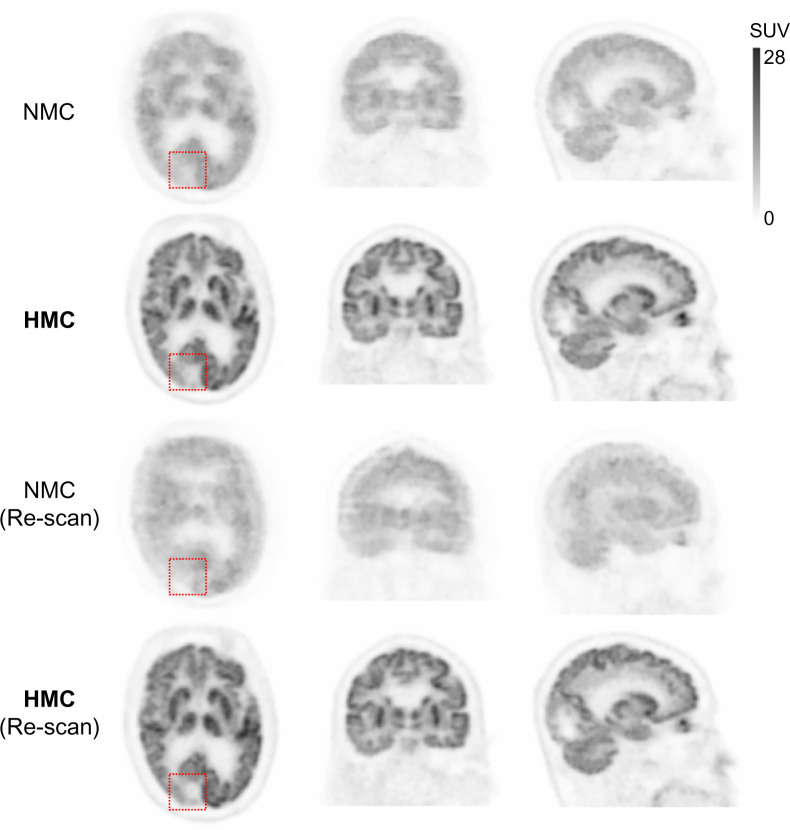
Images corrupted by involuntary HM in both initial PET scan and rescan with NMC. Spatial resolution and contrast were significantly improved after HMC. Dotted box indicates region of hypometabolic edema surrounding suspected metastatic lesion in occipital lobe in patient whose non–small cell lung cancer was revealed after HMC. Injected dose was 314.5 MBq; postinjection first scan and rescan were at 61 and 69 min, respectively; frame duration was 3 min; and body weight was 77 kg. Averaged and maximal HM distance of frontal lobe was 5.0 and 12.6 mm (first scan) and 12.1 and 27.7 mm (rescan), respectively.

Figure 5 features a non–small cell lung cancer patient who exhibited large involuntary HM during both the initial scan and the rescan. The images from these scans without HMC were unsuitable for clinical diagnosis. However, after the application of HMC, both sets of scans showed substantial improvements in resolution and contrast. The images revealed hypometabolic edema surrounding a potential brain metastasis, visible only after HMC application.

For the evaluation studies, Table 3 presents the numeric results of SUV_mean_ changes across different brain regions after HMC. Participants were divided into 2 groups based on the extent of HM: small HM and large HM. This categorization used a 5% threshold in SUV_mean_ change in the frontal lobe after HMC application. In the small-HM category, the mean HM distance was relatively consistent across all brain regions (2.4 mm), with low variability (1.9 mm). Conversely, in the large-HM group, the mean HM distances increased for all ROIs, ranging from 7.3 mm in the cerebellum to 15.0 mm in the frontal region, with a notable increase in both the mean average and variation (10.9 ± 5.9 mm).

**TABLE 3.** SUV_mean_ Change from NMC after HMC for Evaluation Study and Mean HM Distance for Each Brain Region

ROI	Small HM (*n* = 264)		Large HM (*n* = 38)	
	Mean HM distance (mm)	SUV_mean_ change (%)	Mean HM distance (mm)	SUV_mean_ change (%)
Amygdala	2.5 ± 1.9	0.4 ± 2.6	11.8 ± 6.3	19.9 ± 20.8
Caudate	2.6 ± 2.0	0.6 ± 2.6	12.7 ± 7.0	22.9 ± 13.7
Cerebellum	2.1 ± 1.7	−0.2 ± 0.7	7.3 ± 3.9	2.9 ± 3.7
Frontal	2.9 ± 2.0	0.3 ± 1.3	15.0 ± 8.1	12.9 ± 8.3
Hippocampus	2.3 ± 1.8	−0.3 ± 1.4	10.1 ± 5.3	10.6 ± 9.0
Insula	2.6 ± 1.9	0.1 ± 1.1	13.0 ± 7.0	10.0 ± 6.2
Occipital lobe	2.2 ± 1.7	−0.2 ± 0.7	6.7 ± 4.4	2.9 ± 3.9
Parietal lobe	2.4 ± 1.8	−0.0 ± 0.8	9.5 ± 5.2	6.8 ± 6.0
Putamen	2.5 ± 1.9	0.3 ± 1.3	12.4 ± 6.7	12.2 ± 10.4
Temporal lobe	2.5 ± 1.8	0.1 ± 1.0	11.8 ± 6.2	10.0 ± 8.1
Thalamus	2.2 ± 1.8	−0.1 ± 0.8	9.9 ± 5.3	8.2 ± 7.8
Mean average	2.4	0.1	10.9	10.9
SD average	1.9	1.3	5.9	8.9

Data are mean ± SD.

**FIGURE 6.**
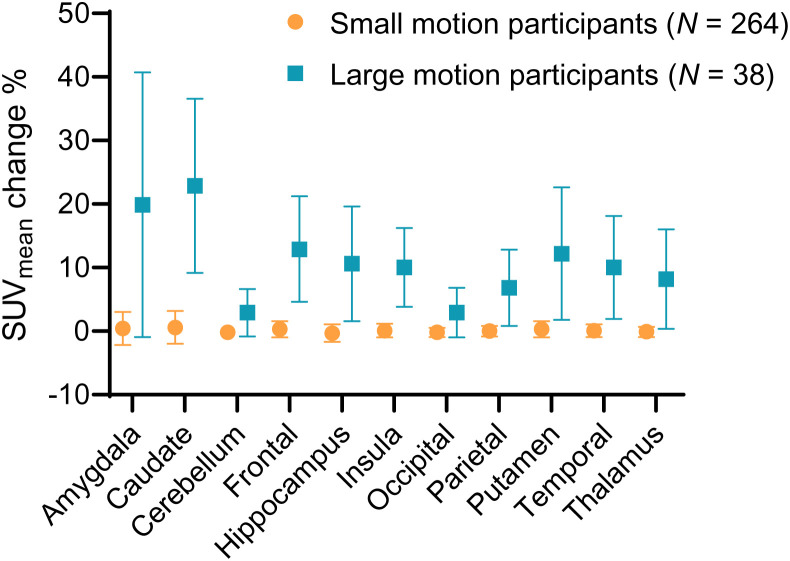
In evaluation studies, SUV_mean_ change at all regions for large-HM group was substantially larger than for small-HM group.

**FIGURE 7.**
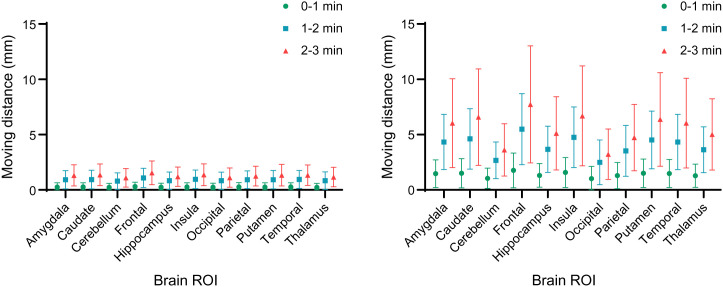
Brain-averaged HM distance across evaluation studies. (Left) Participants (*n* = 268) with small HM amplitude. (Right) Participants (*n* = 38) with large HM amplitude. Averaged HM distances are shown along with time course, that is, 0–1, 1–2, and 2–3 min into PET scan.

Of the 302 participants, 38 experienced large HM, resulting in an average SUV increase of 11% after HMC, whereas the rest showed a minimal uptake increase (0.1%). As depicted in Figure 6, the SUV_mean_ change in the large-HM group was significantly higher than in the small-HM group. The caudate region displayed the most substantial SUV increase, whereas the cerebellum showed the least. Consistent with expectations, Figure 7 reveals that HM amplitude escalated over the course of the scan. For instance, in the large-HM category, the frontal lobe showed increasing HM amplitudes of 2, 5.2, and 7.6 mm at 0–1, 1–2, and 2–3 min into the scan, respectively.

## DISCUSSION

In this study, we conducted both a prospective validation and a retrospective evaluation of an HMC algorithm provided by the uMI Panorama PET/CT scanner. The prospective validation included 15 studies in which participants were instructed to perform HM, and for the retrospective evaluation, 302 clinical brain studies using ^18^F-FDG with a duration of 3 min each were analyzed. Results from the validation study indicated that the HMC algorithm was highly effective, with an average quantitative discrepancy of less than 1% compared with scans without HM. In the retrospective evaluation, it was found that approximately 12% (38/302) of the clinical brain studies exhibited large HM, necessitating the use of HMC. The clinical utility of the HMC algorithm was demonstrated across a variety of brain diseases and clinical scenarios, underscoring its effectiveness in real-world applications. Regarding the efficiency of the HMC, that is, reconstruction time, for the InstrMo studies, it took 11.0 ± 1.1 min to perform the reconstruction using the reconstruction console. All the reconstructions were submitted after the acquisition was finished. For all the evaluation studies, 2.0 ± 9.7 s of data were rejected for each case.

The European Association of Nuclear Medicine guidelines (19) emphasize the importance of neurologic PET imaging for diagnosing cognitive and movement disorders, localizing epileptic foci, detecting neuroinfections such as encephalitis and meningitis, and assessing brain tumors. The guidelines recommend using small-voxel reconstructions to enhance the visualization of brain structures, a method that typically requires longer acquisition times to maintain adequate signal-to-noise ratios. For routine ^18^F-FDG brain scans using a scanner with a short axial field of view, the guidelines suggest acquisition times of 10–15 min per bed position. Non–^18^F-FDG imaging may necessitate even longer times, up to 20–30 min per bed position (19). However, in our study using the uMI Panorama scanner (1), known for its high sensitivity, we completed each clinical scan in just 3 min while preserving sufficient image quality for diagnostic purposes. Notably, within these brief scans, 12% of patients exhibited substantial HM, indicating a potential increase in the need for HMC in longer scans.

The introduction of HMC to PET imaging has significantly improved image quality and brought practical benefits. HMC reduces the need for rescans, decreasing patient waiting times and enhancing comfort by minimizing additional radiation exposure from repeated CT scans. This reduction in rescans also streamlines patient scheduling and workflows, ensuring that appointments run on time, reducing health care providers’ workloads, and allowing for more efficient resource and time management. Additionally, HMC is particularly advantageous for patients who have difficulty remaining still during scans, reducing the need for tight head restraints or sedation and improving patient comfort. The benefits are not only to patients but also to the scanning process, simplifying it and reducing complications from patient HM, ultimately increasing clinical work efficiency.

This paper reviews several significant research efforts in HMC for PET imaging, comparing them with the proposed data-driven method. An alternative approach involves markerless HM tracking using an optical camera, as used by Spangler-Bickell et al. (20), who applied an optical camera attached to the head coil of a PET/MRI system, tracking HM with a large, curved marker on the patient’s forehead. Zeng et al. (21) evaluated a markerless, hardware-based device using stereovision cameras with infrared structured light to capture the patient’s facial surface and create an HM-tracking point cloud. Similar techniques were explored by Olesen et al. and Kyme et al. (22*,*^23^). However, these methods may be compromised by nonrigid facial movements and lack full validation. Another strategy used the Kinect system (24) by Microsoft for monitoring and tracking HM. More recently, Zeng et al. (25) investigated neural networks to predict HM between short frames to expedite HM estimation, though this requires further development for adequate correction accuracy. Additionally, 2 other groups (26*,*^27^) focused on deep learning methods to enhance PET image quality by synthesizing high-count PET images from low-count images, improving registration accuracy. Rezaei et al. (28) proposed computing inertia tensors from time-of-flight back projections for direct estimation of rigid HM parameters. Each method provides a unique perspective and potential solution for addressing HMC challenges in PET brain imaging, underscoring the ongoing and varied research in this critical field.

In concluding our analysis, we acknowledge certain limitations of the current HMC algorithm: its limited applicability to dynamic PET studies, as it is not designed for scenarios requiring tracking of physiologic changes over time; the inability to correct for HM between PET and CT acquisitions, which can result in attenuation mismatch artifacts; and the inability to correct continuous HM, such as tremors in patients with Parkinson disease, because of its frame-based nature. These limitations underscore the need for ongoing improvements and innovation in HMC technology to enhance the accuracy and utility of PET imaging.

## CONCLUSION

We conducted a clinical study with 15 participants using ^18^F-FDG to evaluate the accuracy of the NeuroFocus HMC algorithm for the uMI Panorama PET/CT system. In our validation tests, which included InstrMo, the SUV error after HMC was minimal, averaging −1% ± 3% across all brain ROIs and participants. This was a significant improvement from the −10% ± 5% error observed before applying HMC. In a broader evaluation involving 302 participants, approximately 12% of the short-duration (3-min) clinical brain scans showed substantial HM that required correction. The HMC algorithm effectively corrected HM across various brain diseases, confirming its suitability for clinical brain ^18^F-FDG studies.

## DISCLOSURE

Funding was provided by the National Natural Science Foundation of China (92259304 and 82122033), the Key Program of the Ministry of Industry and Information Technology of the People’s Republic of China (CEIEC-2022-ZM02-0219), and the Shaanxi Province Key Industry Innovation Chain (2022ZDLSF04-12). No other potential conflict of interest relevant to this article was reported.

## Notes

KEY POINTS **QUESTION:** What is the clinical efficacy of the vendor-provided HMC algorithm NeuroFocus for the uMI Panorama PET/CT system, and what is the prevalence of HM in clinical ^18^F-FDG studies? **PERTINENT FINDINGS:** In short-duration (3-min) clinical brain ^18^F-FDG studies, approximately 12% of patients experienced substantial HM that necessitated the use of HMC. NeuroFocus effectively corrected for HM, and its accuracy was deemed adequate for clinical applications. **IMPLICATIONS FOR PATIENT CARE:** NeuroFocus has the potential to enhance the quality of brain PET images. It can reduce the clinical necessity for rescanning patients due to HM, thereby improving the patient experience and streamlining the diagnostic process.
